# Mental health treatments in an Italian prison: the Parma integrated approach

**DOI:** 10.1192/j.eurpsy.2022.225

**Published:** 2022-09-01

**Authors:** L. Pelizza, D. Maestri, G. Paulillo, P. Pellegrini

**Affiliations:** AUSL di Parma, Department Of Mental Health, Parma, Italy

**Keywords:** Psychiatric services, rehabilitation, Prison, mental health care

## Abstract

**Introduction:**

Mental health interventions for Italian (and European) prisoners with mental disorders remain a problematic issue, despite radical changes in general psychiatric care and a 2008 major government reform transferring mental health care in prison to the National Health Service. Indeed, according to the American Psychological Association, 64% of incarcerated individuals report mental health concerns.

**Objectives:**

The aim of this study is to describe the mental health intervention model implemented since January 2020 for prisoners allocated in the Parma Penitentiary Institutes (PPI). This approach is specifically based on specialized, “person-centered” and “person-tailored” therapeutic-rehabilitation plans in line with psychiatric treatments usually provided in community mental health-care centers of the Parma Department of Mental Health.

**Methods:**

All the processes and procedures included in the PPI intervention model were first carefully described, paying special attention to the service for newly admitted prisoners and each typology of specialized therapeutic-rehabilitation treatment potentially provided. Additionally, a preliminary descriptive process analysis of the first six months of clinical activity was also performed.

**Results:**

Since January 2020, 178 individuals entered the PPI service for newly admitted prisoners. In total, 83 (46.7%) of them were engaged in the services of the PPI mental health-care team (35 with pathological addiction and 48 with mental disorders): 56 prisoners were offered an integrated mental health intervention and 27 exclusively an individual psychological or psychiatric treatment.

**Conclusions:**

The results support the potential applicability of an integrated mental health intervention in prison, planning a person-tailored rehabilitation in close collaboration with the prisoners, their families and the local mental health/social services.

**Disclosure:**

No significant relationships.

